# Behavioral mapping of children’s physical activities and social behaviors in an indoor preschool facility: methodological challenges in revealing the influence of space in play

**DOI:** 10.1186/s12942-019-0191-y

**Published:** 2019-11-20

**Authors:** Ajoke R. Onojeghuo, Candace I. J. Nykiforuk, Ana Paula Belon, Jane Hewes

**Affiliations:** 1 School of Public Health, University of Alberta, Edmonton, T6G 1C9 Canada; 2 Department of Obstetrics & Gynecology, Faculty of Medicine & Dentistry, University of Alberta, Edmonton, T6G 2S2 Canada; 3 Faculty of Education and Social Work, Thompson Rivers University, Kamloops, BC V2C 0C8 Canada

**Keywords:** Free play, GIS, Social behaviors, Gridding, Indoor environments, Physical activity

## Abstract

**Background:**

GIS (Geographic Information Systems) based behavior maps are useful for visualizing and analyzing how children utilize their play spaces. However, a GIS needs accurate locational information to ensure that observations are correctly represented on the layout maps of play spaces. The most commonly used tools for observing and coding free play among children in indoor play spaces require that locational data be collected alongside other play variables. There is a need for a practical, cost-effective approach for extending most tools for analyzing free play by adding geospatial locational information to children’s behavior data collected in indoor play environments.

**Results:**

We provide a non-intrusive approach to adding locational information to behavior data acquired from video recordings of preschool children in their indoor play spaces. The gridding technique showed to be a cost-effective method of gathering locational information about children from video recordings of their indoor physical activities and social behaviors. Visualizing the proportions of categories and observed intervals was done using bubble pie charts which allowed for the merging of multiple categorical information on one map. The addition of locational information to other play activity and social behavior data presented the opportunity to assess what types of equipment or play areas may encourage different physical activities and social behaviors among preschool children.

**Conclusions:**

Gridding is an effective method for providing locational data when analyzing physical activities and social behaviors of preschool children in indoor spaces. It is also reproducible for most GIS behavior mapping focusing on indoor environments. This bypasses the need to have positioning devices attached to children during observations, which can raise ethical considerations regarding children’s privacy and methodological implications with children playing less naturally. It also supports visualizations on behavior maps making them easier to interpret.

## Background

Free play encourages children to be self-driven in play using their own imagination in unregulated play while developing social behaviours and physical literacy levels that influence their long-term health [1, 2]. Features of these play spaces, such as the types of play equipment available and size, have been linked to increased physical activity levels and a variety of play behaviors among preschool children [3, 4]. Maps provide the opportunity to visualize information gathered about play spaces and how they are utilized during children’s play activities or how they may encourage certain social behaviors. Behavior mapping (also known as behavioral mapping) affords researchers the opportunity to gather, process, analyze, and represent data in efficient ways making it easier to determine how the environment may influence certain behaviors [5]. It relies on direct observation of behaviors and a map of the environment where behaviors are recorded, analyzed, and displayed [6].

Behavior mapping allows for effective representation of geo-located activities and serves as an effective tool to help interpret behavioral patterns [6, 7] even when the audience is not familiar with the subject [8]. The addition of precise locational information to behavior data allows for accurate mapping of place-dependent behaviors and activities in these environments. Creating a GIS (Geographic Information System) of physical activities and social behaviors underpins the correct positioning of observed behaviors and activities and their visualization on a behavior map. GIS is an effective tool for acquiring or creating locational data, storing, and linking such data with observational data (such as that for physical activities and social behaviors). It has been applied in several fields, including public health and community medicine, to effectively combine and analyze vast data collected geospatially [9–12]. GIS-based behavior maps can show how frequently an activity occurred at a location and how other activities relate to it in time and space [7].

One method of creating locational information involves the use of existing paper maps or electronic devices to sketch locational information of subjects or an event during observation [5, 13–15]. These locations are often estimated based on the quick real-time perception of the observer [14, 16], which may lead to locational errors [17]. Other indoor positioning methods involve the use of radio and infra-red waves which may be harmful to growing children [18, 19], Wi-Fi (Wireless Fidelity) triangulation, and magnetic positioning [20]. Geographic Position Systems (GPS) are the most commonly used to capture locational data alongside other variables, such as social behaviors, travel time and play activities [5, 7, 21, 22]. The positional error of the GPS which could be about 3 m [23], is negligible for outdoor environments or large regions of interest such as neighborhoods, municipalities, or countries [24–28] but increases significantly in indoor environments [29]. Although precise in some cases, some of these positioning methods can be cost-prohibitive for academic research and raise some ethical issues such as the children’s rights to privacy and a reduced sense of independence [30].

Alternatively, in small areas such as indoor spaces, spatial measurements can be collected using a local grid laid out in the area as long as the two axes (X and Y) used are at right angles on a flat plane [31]. Gridding enables the creation of locational information where it may not be possible to use the GPS. Grids are often used to determine the position of a particular thing, place, or activity in space. They are also used for point data and geographic information aggregation in research fields such as in assessing children’s physical activities and behavior [32, 33] and the linguistic landscape of a city [34]. Some studies have used raster-based grids for positioning when measuring physical activity [23]. Gridded digital maps have also been used real-time to code the location of children in their play environment [15, 32]. However, unlike video recordings, the real-time approach to collecting data leads to the likelihood of human coding errors [35] and leaves no room for future re-observation of the scenarios where coding occurred. Most of the aforementioned tools and methods for positioning require that locational information be assigned or collected real-time while other required datasets are collected.

This research looked at the dynamics of play in an indoor preschool play space using data collected from videos and applied GIS. At the time of video data collection for the initial phase of this project, no indoor locational information compatible with a GIS was acquired for the preschool children. We combined data collected using fixed camera systems with validated layout (base) maps and gridding techniques to provide locational information for the video-recorded observations in this study. The purpose of this secondary analysis was to create a low cost, small scale GIS for better assessment of how indoor the preschool environment shapes children’s play, while improving the visual representation of the physical activity levels and types and social behaviors of children. Such techniques could also be extended for future longitudinal studies of children’s play. A descriptive and short-term temporal analysis of play variables in this indoor play space is also presented to illustrate the advantages of using a small scale GIS retrospectively.

## Methods

### The Love To Play project and study area

The municipal department of Recreation, Parks, and Culture of Strathcona County, in Canada, set up a purposive free-play space in the rural recreation facility of Ardrossan Recreation Complex (ARC) in 2014 in response to the finding that 21% of rural Strathcona County children were struggling in developmental areas of communication, general knowledge and emotional maturity [2]. A community-university partnership project was conceived to evaluate how preschool children (aged three to five) utilized preschool spaces relative to developmental areas associated with play in that facility and two comparable sites (more information about this project can be found elsewhere [36]). The project was approved by the University of Alberta Research Ethics Board. The project focused on three facilities (one urban and two rural) providing free-play-based preschool programs in Strathcona County. One of rural facilities was the ARC.

The free-play-based preschool program at the ARC is offered in an innovative play space, which allowed the research team to investigate how play space design affects children’s play activities and social behaviors [36]. This space consists of two major rooms: a preschool room for structured activities and the Love To Play (LTP) room for unstructured, free play. The LTP room features fixed play equipment, such as: the wind tube experiment where children feed the tubes with soft balls and scarves and watch them travel; the ball experiment; a ball lift; a magnet board; a grocery till with cash registers and grocery store shelf with store items; puppet boxes; and a wood tree house. It also provides loose parts (portable play equipment) such as large rubber blocks, shopping carts, and skipping pods which are light enough for the children to move around the room. Videos collected from the LTP room during free play served as the basis for the small scale GIS shown in this research.

### Video data collection and the identification of children

The videos of children’s play were recorded on one weekday in each month in the mini-gyms or LTP Room and in the preschool rooms at the 3 preschool facilities covered in the original project. These preschools were only open a few days a week and not Monday to Friday. Our program partners limited data collection to 1 day per month to limit burden on the preschool staff. Further, two of these facilities were in rural areas that were not in close proximity to the universities involved, and required some travel planning to coordinate visits across sites. The same cameras had to be set up and re-used at the three different preschool facilities to keep expenditure within the budget determined by the grant funding for the project. The monthly dates for video collection were selected based on the day(s) of the week when these preschool facilities were open and care was taken to ensure no overlap in dates scheduled for the different facilities.

**Fig. 1:**
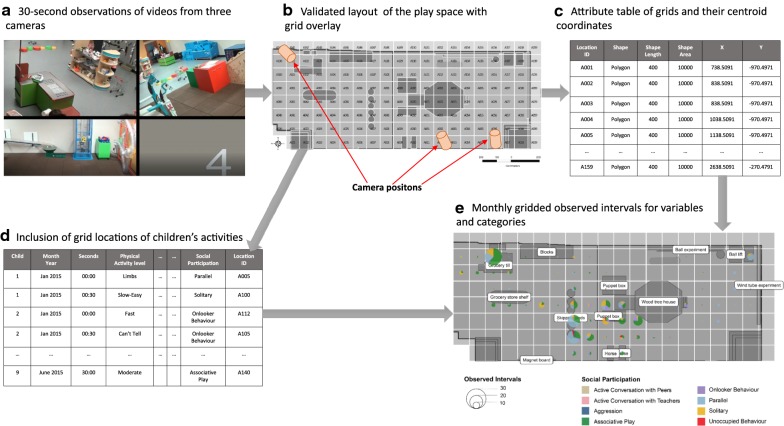
Steps for the creation of locational information and the connection between grids and modified OSRAC-P coded data for behavior mapping

A GIS or equipment-based analysis was not the focus of the initial phase of this research at the time of data capture. Therefore, no prior research strategy was designed for collecting detailed locational information beyond the scope of the coding algorithm used. The attachment of locational information for a small scale GIS was conceived during a secondary analysis of data collected in the LTP room. Figure 1 shows the process for creating locational information from video recordings and then merging this with tables of coded play and social behavior variables. The different segments shown in this figure are discussed further in subsequent sections of this paper.

Three video cameras were positioned in the room to capture children’s play from different view angles for 30 min in each month between September 2014 and June 2015 (Fig. 1). They were set up on the walls at locations high enough to ensure that the children were not aware of the presence of the cameras and movement and play activities of children were not hampered. The cameras were positioned over areas where the children played mostly. Their positions minimized any potential influence of the cameras on the children’s play activities and maximize the area covered by each camera. The three videos for each observation period were combined in a three-way split-screen to improve the manual identification of randomly selected children in the room and their activities. The videos were clear enough to identify the children in the room and distinguish them apart.

Nine children (60% of the registered number of children per term) were randomly selected from the videos monthly. The selected children were assigned child identification numbers in the order they were first spotted in the videos. Identifiable features of the children for each split screen video combination were documented to ensure they were easily identified throughout the video. Such features including hairstyles, hair color/length, clothing, camera where they are best identified, and activities when first spotted were documented. An example would be “child 8 (first spotted at 08 s) in January had brown hair, wore a pink shirt, yellow pants and was standing behind the grocery till holding a yellow bottle”. When needed, extra efforts were taken to distinguish children who appeared similar by watching these videos repeatedly before analyzing and coding them. These nine children were then followed throughout the room for the duration of the videos while considering their unique features.

### Coding play activities and play behaviors

The play activities and social behaviors of the children in the room were coded from the videos using a modified version of the Observational System for Recording Physical Activity in Children-Preschool version (OSRAC-P). The OSRAC-P coding tool [37] uses a transient time sample scheme (i.e., 30-s intervals with 5-s observation and 25-s coding) to collect observational information about the type and level of physical activity, location, the play context (indoor and outdoor), initiator of activity, group composition, and prompts during physical activities among preschool children [37, 38]. It does not include the indoor positions of children during activities in a defined space and only specifies the location as “indoor”, “transition”, or “outdoor” area. The modified OSRAC-P system [36, 38] used in this study incorporates the observation of social participation (a type of social behavior) in free play. The modified OSRAC-P coding system consists of eight variables: physical activity level; physical activity type; physical location; play activity; initiator of activity; group composition; prompt for physical activity; and social participation. The categories of social participation were adapted from social behaviors described in the Play Observation scale [39, 40] and the Social Play Continuum [41], including codes for “Active Conversation with a Peer”, “Active Conversation with a Teacher”, “Aggression”, “Associative Play”, “Cooperative Play”, “Imitation”, “Onlooker Behaviour”, “Parallel”, “Unoccupied Behaviour”, “Solitary” and “Can’t Tell”. A supplementary table showing the different types of social participation and the descriptions of each category is attached as supplentay information (Appendix). Non-applicable variables of the original OSRAC-P system such as the “outdoor/gym education/play context” were not considered during coding as the activities studied all occurred indoors. The modified OSRAC-P is available upon request from the corresponding author. The 30-min split-screen videos were coded at 30-s intervals (60 intervals for each three-way split-screen video). For this present study, only the videos from the months of January to June 2015 were analyzed, which resulted in a total of 360 time intervals for analysis.

### Creating locational information

The first step in behavior mapping is the creation of a map of the space being analyzed [42] or the provision of an existing (but not necessarily up-to-date) map as a guide [43]. In this case, the layout plan of the LTP room provided by the managers of the preschool programs run by Strathcona County was digitized using the ArcGIS 10.5 software [44]. The shapefiles created from the digitized layout map served as a base for customizing the locations of equipment in the LTP room seen on the videos monthly for use in the GIS analysis.

A validation exercise was carried out by three trained research team members to confirm the dimensions of the LTP room and the locations of fixed equipment seen on the digitized layout plan. Sample linear measurements and photographs were taken and compared to the dimensions shown on the digitized layout map. This was to ensure the adequacy of the gridding approach chosen in identifying and coding children at various locations in the LTP room. During the validation exercise, it was observed that the dimensions of some of the fixed equipment in the room were not accurately depicted on the digitized layout map. The distances between equipment attached to the wall (like the magnet board and ball experiment) and fixed equipment in the middle of the room were measured to fix other features at correct locations on the digitized LTP room layout. Another significant feature of the LTP room that was important in validating positions was the presence of floor tiles which were 50 cm × 50 cm. Counting the number of floor tiles along a line between walls provided perspective for locating features in the room.

A trial coding of the locations of children in the LTP room was done using different grid shapes and sizes. Some of these grids were irregular grids around fixed equipment, a regular 100 cm × 100 cm grid, and a regular 50 cm × 50 cm grid. The 50 cm × 50 cm grids were too small to position the children during 30-s intervals and the irregular grids did not capture the locations of children who were not playing around fixed equipment during observed intervals. After considering the 3 grid sizes, a 100 cm × 100 cm grid resolution was selected for positioning children in the room. The validated layout map of the LTP room was gridded at 100 cm × 100 cm resolution and unique grid identification codes were assigned to each grid square. The grid cells were created using the Fishnet tool in ArcGIS 10.5 as a sequence of polygons. Each 100 cm × 100 cm grid cell was estimated to be the same size as four 50 cm × 50 cm floor tiles stacked together to form a square in the LTP room. The coordinates of the centroids of the grid polygons were extracted using the language, statistical software “R” [43]. These centroids were used to position the charts generated for each grid polygon. The database of coded observations of play activities and social behaviors were merged with shapefiles of the monthly equipment locations using the grid identification codes.

Most of the GIS analysis including merging the locational data with other modified OSRAC-P variables were done using the programming language, statistical software “R” [45] version 3.5.1 and its vast packages especially “dplyr” [46] GGPLOT2 [47] and “scatterpie” [48]. “R” allows for robust manipulation of databases and geospatial data from a variety of sources with good facilities for merging these data. The monthly videos recorded in the LTP room were re-analyzed by a trained research team member to assign locations of children for all 30-s intervals (following the OSRAC-P convention) using the unique grid names from the layout map. The locational information was added to databases containing the other coded variables.

**Fig. 2:**
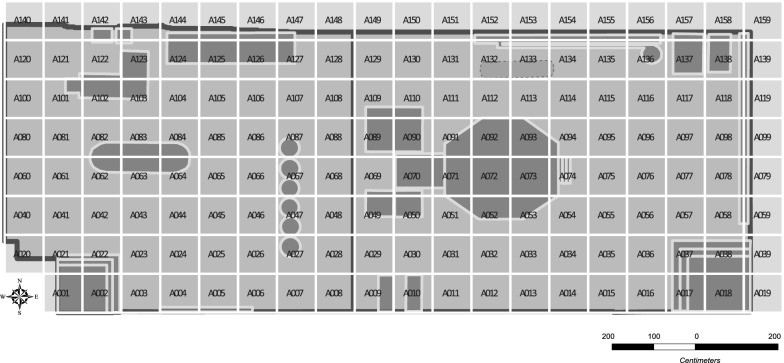
The LTP room equipment layout for June 2015

At the start of the location coding exercise, the videos showed that the layout of some equipment in the room (such as the grocery till) were different for all months. However, the positions of fixed equipment like the ball lift, ball experiment, wind tube experiment, wood tree house, and magnet board were the same for the 6 months analyzed. The LTP room layout was changed regularly to encourage new play behaviors, stimulate imagination and creativity, and avoid boredom. The videos also revealed that, in addition to fixed equipment provided, the children play around certain areas in the LTP room using portable play equipment, such as rubber blocks and skipping pods. These loose pieces of play equipment were also positioned in the room differently each month and were not present through all the months considered. New shapefiles showing the actual positions and orientations of equipment each month were created to ensure play activities were represented relative to equipment and open spaces correctly. The grid locations were uniform monthly with the same cell names. Different layout maps of the LTP room were generated for each month and then used to position the children monthly. Figure 2 shows the layout of the LTP room in June 2015 after the equipment were properly positioned and the grids overlaid.

### Data visualization and grid aggregation

The observed intervals for each month were summed up for each grid cell. The individual categories for each variable were plotted on unique grid cells. The behavior maps generated in this format were too many to process visually at a glance with some variables such as physical activity type having more than ten maps to cover all categories. The differences in observed intervals between categories of play variables were not distinct or comparable visually from these maps. Therefore, grid-based pie charts to present percentages were considered for each variable each month to improve the visualization of play activities and behaviors. Bubble pie charts [51] were used to represent the categories of the variables on a single grid map to allow for the representation of observed intervals in addition to percentages. This was to preview the distribution of the categories within these variables for each grid cell and reduce the number of maps required to present results effectively. These bubble pie charts were positioned using monthly equipment shapefiles and the general grid centroids.

The grid-based visualization using bubble pie charts also served as the basis for the aggregation of grid cells to analyze activities around each piece of equipment or play area alongside the videos. To aid longitudinal analysis using similar monthly geospatial locations for all equipment and play areas, a uniform naming scheme was adopted to aggregate grid cells relative to each equipment or play area for each month. The uniform naming scheme does not imply that the same set of grids were aggregated for each equipment or play area monthly. For example, “Wind tube experiment” refers to locations where all observed play occurred using the wind tube experiment. A uniform grid cell aggregation approach could not be adopted for all months because the location of some movable equipment in the LTP room varied monthly. The monthly videos showed when observed play activities and social behaviors were related to a piece of equipment directly or indirectly. For example, children would stand closer to the wood tree house rather than the wind tube while waiting to catch the scarves and soft balls pushed out of the wind tube. The videos also showed when play activities were in an open area being utilized collectively by more than one child, or at varied locations in the room by individual children.

**Fig. 3:**
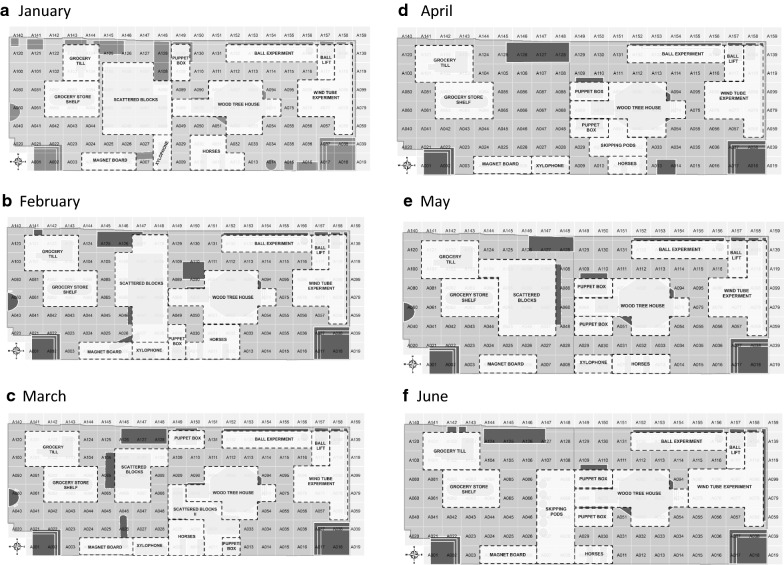
Definition of boundaries for grid cell aggregation for all equipment or play areas in the LTP room from January to June 2015

Table 1 shows the equipment or play area names adopted and the corresponding areas covered. “Scattered blocks”, “Scattered blocks II”, and “Skipping pods” refer to play areas where those portable play equipment were used by the children. Locations where children played solitarily or away from these named areas were not aggregated. Figure 3 presents the grid cells aggregated around equipment or play areas in the LTP room monthly.

**Table 1:** Definition of grid areas for each equipment/play area in the LTP room

Equipment/play area	Associated area
Ball experiment	The ball experiment and all areas with play directly related to the ball experiment
Ball lift	The ball lift and all adjoining areas with play directly related to the ball lift
Grocery store shelf	Location of the grocery store shelf or where play activity was related to the grocery store shelf directly
Grocery till	Location of the grocery till or play activity observed was related to the grocery till directly
Horses	The area where play horses are located or where play activity was related to the horses
Magnet board	The area where the magnet board is located or where the play activity was related to the magnet board
Puppet box	Location of puppet box or area where the play activity observed was related to the puppet box
Scattered blocks	An open area with a focused activity using blocks located between the grocery till, grocery store shelf, and the wood tree house
Scattered blocks II	An open area with a focused activity using blocks located between the wood tree house and the horses
Skipping pods	An open area with skipping pods arranged inclusive of areas where the play activity was skipping pod related
Wind tube experiment	The wind tube experiment and all areas of play directly related to the wind tube experiment
Wood tree house	Location of the wood tree house, steps/slides leading in, or area where the play is wood tree house-related directly.
Xylophone	The area where xylophone is located or where the play activity was related to the xylophone directly

### Statistical analysis

The OSRAC-P physical activity levels were recategorized as follows: sedentary (combines limbs and stationary activity), light (slow/easy activity) and moderate/vigorous (combines moderate and fast activities. The data for physical activities and social behaviors around each play equipment or area was collated and analyzed using R [45]. The observed intervals were summed up monthly for each physical activity and social behavior category and then converted to percentages per month around each equipment or play area. For the descriptive analysis, the mean and standard deviation (SD) of the monthly percentages of each category were used to summarize the physical activities and social behaviors.

## Results

### Behavior maps

**Fig. 4:**
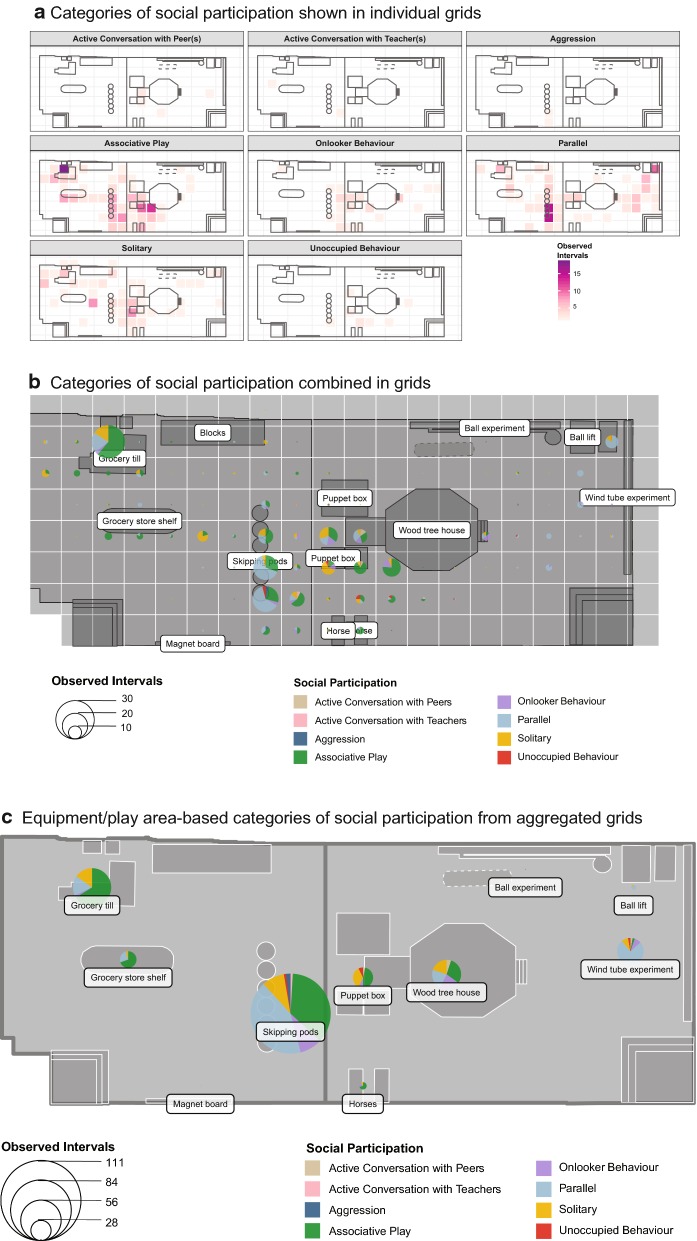
Visualization of social participation in June 2015 using 3 different types of GIS behavior maps. **a**, **b** represent the same data while **c** shows aggregates around major equipment/play areas in the room

The visualization results presented are samples of the outcomes of the methods used including gridding. Given social participation had a high number of categories, the maps for this variable are included here to illustrate the differences apparent when visualizing the results in different ways on the layout of the room. Figure 4a shows the observed intervals for all categories of social participation displayed on individual grids and maps. This visualization of social participation had eight maps (one map for each category of social participation) for June 2015. The figure shows that the dominant social participation type around the grocery till was associative play while parallel play occurred more around skipping pods in. However, it is difficult to compare less obvious observed intervals of some social participation types such as aggression and unoccupied behavior around equipment in the LTP room. Figure 4b shows the distribution of social participation types in the LTP room in June 2015 displayed using grid-based bubble pie charts. This behavior map fuses all the categories of social participation together in one map. When compared to Fig. 4a, it is easier to observe that associative play was dominant around the grocery till in Fig. 4b, while parallel play occurred more around the skipping pods. The sizes of the bubble pie charts show the relative proportions of all categories of social participation and the total observed intervals per grid cell. Figure 4c presents a visualization of aggregated grids to present activities around unique equipment or play areas in a simplified manner, which aided a better interpretation of the behavior maps. For example, the skipping pods were the most popular equipment that the children played with collectively in June 2015. One can also detect that around the puppet boxes, most children were either involved in associative play or were solitary.

### Equipment and play area statistics

The equipment or play-area based analysis showed higher observed intervals for girls (77.57 ± 12.83%) around the grocery store shelf than boys (35.36 ± 33.36%). However, higher observed intervals of boys than for girls (Table 2) occurred around portable play equipment such as the scattered blocks (93.73 ± 6.30% vs. 8.35 ± 5.78%), scattered blocks II (67.90% vs. 32.10%), skipping pods (72.30 ± 31.53% vs. 27.70 ± 31.53%), and wind tube experiment (61.79 ± 29.95% vs. 45.85 ± 26.14%). This sex-based difference was not distinct around the wood tree house (50.21 ± 29.79 for girls and 58.16 ± 33.62 for boys) and the grocery till (53.55 ± 32.70 for girls and 55.37 ± 36.52 for boys).

**Table 2:** Sex-based percentages around equipment or play areas (mean (SD))

Equipment or play area	Sex	
	% Girls	% Boys
Ball experiment	29.35 (41.35)	75.54 (38.88)
Ball lift	43.75 (29.95)	63.54 (32.20)
Grocery store shelf	77.57 (12.83)	35.36 (33.68)
Grocery till	53.55 (32.70)	55.37 (36.52)
Horses	58.02 (35.70)	34.99 (36.24)
Magnet board	28.60 (42.02)	76.16 (39.35)
Puppet box	36.92 (34.85)	52.56 (40.43)
Scattered blocks	8.35 (5.78)	93.74 (6.30)
Wind tube experiment	45.85 (26.14)	61.79 (29.95)
Wood tree house	50.21 (29.79)	58.16 (33.62)
Xylophone	0 (0)	80 (44.72)
Scattered blocks II	32.10 (0)	67.90 (0)
Skipping pods	27.70 (31.53)	72.30 (31.53)

The highest mean moderate/vigorous physical activity levels (Table 3) were seen around the xylophone. The xylophone had the lowest observed intervals (eight intervals) for the 6-month period analyzed. Considering the observed intervals around each equipment or play area, moderate/vigorous physical activity was observed mostly with play involving the use of portable play equipment. Moderate/vigorous levels were between 10.06 ± 7.35% with scattered blocks, 11.11% around scattered blocks II (81 intervals), and 13.06 ± 18.47 with the skipping pods (137 intervals). The only fixed equipment with similar levels of moderate/vigorous activity levels was the wind tube experiment (10.47 ± 10.65%). The highest levels of light physical activity were observed with the scattered blocks (55.58 ± 18.09% with the scattered blocks and 72.84% for scattered blocks). Sedentary physical activity levels were the most observed in the room. Sedentary activities aroud the ball lift declined between January and June 2015 while light activity levels increased monthly.

**Table 3:** Percentages of physical activity levels around equipment or play areas (mean (SD))

Equipment or play area	Physical activity level		
	% Sedentary	% Light	% Moderate/vigorous
Ball experiment	54.12 (31.21)	40.32 (31.91)	5.56 (11.44)
Ball lift	72.05 (15.82)	25.45 (14.10)	2.49 (4.49)
Grocery store shelf	54.22 (12.35)	43.71 (12.62)	2.08 (3.35)
Grocery till	72.02 (12.89)	26.12 (14.35)	1.87 (3.37)
Horses	52.97 (39.56)	26.90 (26.73)	3.46 (7.29)
Magnet board	70.59 (30.12)	28.37 (29.37)	1.04 (2.55)
Puppet box	50.83 (29.45)	28.53 (19.32)	3.97 (6.85)
Scattered blocks	34.37 (19.29)	55.58 (18.09)	10.06 (7.35)
Wind tube experiment	50.05 (13.85)	39.49 (10.94)	10.47 (10.65)
Wood tree house	56.76 (21.72)	37.49 (17.43)	5.75 (6.73)
Xylophone	20 (27.39)	46.67 (36.13)	13.33 (29.81)
Scattered blocks II	16.05 (0)	72.84 (0)	11.11 (0)
Skipping pods	48.82 (39.74)	38.12 (21.27)	13.06 (18.47)

For social participation (Table 4), associative play in the room was highest around the grocery till (60.51 ± 23.08%) and the horses (51.65 ± 35.53%). Associative play was the most observed social participation around the scattered blocks (34.44 ± 26.98%). Social participation around the xylophone, which had the smallest observed intervals, was mostly solitary (53.33 ± 50.55%). The ball experiment, magnet board, and wood tree house also had high percentages of solitary play (42.78 ± 45.34%, 45.30 ± 45.67%, and 43.16 ± 25.82% respectively). Parallel play showed high percentages around fixed equipment like the ball lift (40.60 ± 29.89%), grocery till (44.84 ± 24.39%), and the wind tube experiment (42.87 ± 28.71%). When compared to other equipment/play areas, the puppet box was a hot spot for unoccupied behavior (7.33 ± 9.27%).

**Table 4:** Percentages of social participation types around equipment or play areas (mean (SD))

Equipment or play area	Social participation				
	Active conversation with teachers	Associative play	Onlooker behaviour	Parallel	Solitary
Ball experiment	0 (0)	40.57 (44.32)	4.73 (6.02)	11.92 (13.08)	42.78 (45.34)
Ball lift	1.85 (4.54)	15.95 (19.52)	4.99 (5.60)	40.60 (29.89)	34.76 (17.64)
Grocery store shelf	0 (0)	30.74 (29.47)	3.87 (5.19)	44.84 (24.39)	19.24 (14.89)
Grocery till	0 (0)	60.51 (23.08)	0.61 (0.94)	18.34 (9.20)	14.93 (18.07)
Horses	0.85 (2.09)	51.65 (35.53)	7.84 (9.34)	7.59 (7.66)	14.54 (16.43)
Magnet board	0 (0)	26.84 (34.33)	3.12 (7.65)	24.73 (38.69)	45.30 (45.67)
Puppet box	1.59 (3.89)	28.93 (30.25)	16.36 (28.98)	14.15 (20.18)	13.68 (15.18)
Scattered blocks	0.56 (1.11)	34.44 (26.98)	7.36 (5.04)	22.74 (17.89)	26.81 (18.82)
Wind tube experiment	0 (0)	30.55 (19.73)	4.44 (4.21)	42.87 (28.71)	20.23 (21.28)
Wood tree house	0.83 (2.04)	23.00 (25.00)	9.00 (10.85)	22.45 (10.84)	43.16 (25.82)
Xylophone	0 (0)	20 (44.72)	0 (0)	6.67 (14.91)	53.33 (50.55)
Scattered blocks II	6.17 (0)	38.27 (0)	12.35 (0)	28.40 (0)	9.88 (0)
Skipping pods	0.45 (0.64)	31.48 (6.44)	6.43 (3.65)	30.79 (16.34)	27.58 (26.27)

Equipment or play area	Social participation				
	Unoccupied behaviour	Active conversation with peers	Aggression	Imitation	
Ball experiment	0 (0)	0 (0)	0 (0)	0 (0)	
Ball lift	1.85 (4.54)	0 (0)	0 (0)	0 (0)	
Grocery store shelf	1.30 (2.09)	0 (0)	0 (0)	0 (0)	
Grocery till	3.28 (5.45)	0.57 (1.28)	2.78 (5.56)	0 (0)	
Horses	0 (0)	1.03 (2.29)	0 (0)	0 (0)	
Magnet board	0 (0)	0 (0)	0 (0)	0 (0)	
Puppet box	7.33 (9.27)	1.55 (2.13)	0 (0)	0 (0)	
Scattered blocks	5.31 (4.42)	2.65 (2.51)	0.56 (0.49)	0.74 (1.05)	
Wind tube experiment	0.45 (1.10)	0.78 (1.19)	0.90 (1.81)	0.60 (0.85)	
Wood tree house	0.72 (1.77)	1.00 (2.24)	0 (0)	0 (0)	
Xylophone	0 (0)	0 (0)	0 (0)	0 (0)	
Scattered blocks II	2.47 (0)	1.23 (0)	1.23 (0)	0 (0)	
Skipping pods	2.37 (2.08)	0 (0)	1.80 (0)	0 (0)	

Most of the children who played with fixed equipment in the room stood around them (Table 5). The highest percentages of pushing or pulling were observed around the grocery store shelf (18.90 ± 12.93%) and around the ball experiment (16.67 ± 40.82%). Rocking occurred mostly around the scattered blocks II area (16.67 ± 40.82%). This area in the room was close to the location of the horses. Lifting/carrying constituted 14.68 ± 9.11% of the physical activity types observed with the scattered blocks. The highest percentages of pushing/pulling were seen around the ball experiment (16.67 ± 40.82%) and the grocery store shelf (18.90 ± 12.93%).

**Table 5:** Percentages of physical activity types around equipment or play areas (mean (SD))

Equipment or play area	Physical activity type							
	Climb	Crawl	Hit/pound	Jump/skip	Lie down	Lift/carry	Push/pull	Run
Ball experiment	0 (0)	0 (0)	0 (0)	2.86 (6.39)	0 (0)	0.76 (1.86)	16.67 (40.82)	0.79 (1.94)
Ball lift	0 (0)	0 (0)	0 (0)	0 (0)	0 (0)	2.38 (5.83)	0 (0)	1.85 (4.54)
Grocery store shelf	0 (0)	0 (0)	0 (0)	0 (0)	0 (0)	2.57 (4.14)	18.90 (12.93)	0 (0)
Grocery till	2.78 (6.80)	0 (0)	0 (0)	0.92 (1.32)	0 (0)	1.38 (1.65)	5.97 (4.27)	3.72 (6.49)
Horses	6.51 (11.70)	0.51 (1.15)	0 (0)	0 (0)	0.43 (1.05)	0.43 (1.05)	0 (0)	3.03 (7.42)
Magnet board	0 (0)	1.72 (2.73)	0 (0)	2.50 (5.59)	0 (0)	0 (0)	0 (0)	0 (0)
Puppet box	1.29 (2.01)	2.50 (5.59)	1.04 (2.08)	0.71 (1.60)	2.47 (3.83)	1.28 (3.14)	0.60 (1.46)	3.27 (3.93)
Scattered blocks	0.68 (1.35)	1.23 (1.44)	0.91 (1.27)	1.81 (2.37)	0.23 (0.46)	14.68 (9.11)	3.30 (5.04)	4.25 (3.06)
Wind tube experiment	0 (0)	1.05 (1.06)	0 (0)	8.11 (7.86)	0 (0)	1.26 (2.06)	0.99 (1.54)	7.76 (6.66)
Wood tree house	5.22 (5.80)	1.75 (2.74)	0 (0)	3.79 (3.76)	2.80 (4.54)	0.72 (1.77)	2.54 (2.79)	1.56 (2.42)
Xylophone	0 (0)	0 (0)	8.33 (16.67)	25.00 (50)	0 (0)	0 (0)	0 (0)	6.67 (14.91)
Scattered blocks II	0 (0)	4.94 (0)	1.23 (0)	6.17 (0)	1.23 (0)	7.41 (0)	2.47 (0)	2.47 (0)
Skipping pods	0 (0)	0 (0)	0 (0)	22.52 (0)	0 (0)	1.80 (2.55)	1.35 (1.91)	9.58 (8.11)

Equipment or play area	Physical activity type							
	Sit/squat	Stand	Walk	Throw	Ride	Rock	Roll	Stand
Ball experiment	46.40 (28.33)	7.72 (12.24)	25.28 (18.79)	0 (0)	0 (0)	0 (0)	0 (0)	0 (0)
Ball lift	19.50 (19.81)	51.92 (31.13)	23.71 (11.66)	0 (0)	3.85 (0)	0 (0)	0 (0)	0 (0)
Grocery store shelf	0.98 (2.40)	53.24 (13.24)	24.31 (20.57)	0 (0)	0 (0)	0 (0)	0 (0)	0 (0)
Grocery till	8.77 (8.98)	61.63 (17.88)	14.99 (11.65)	0 (0)	0 (0)	0 (0)	0 (0)	0 (0)
Horses	33.69 (28.19)	18.86 (20.48)	15.70 (28.49)	0 (0)	5.13 (0)	6.84 (11.84)	0 (0)	0 (0)
Magnet board	10.51 (9.59)	60.08 (34.82)	19.65 (17.94)	0 (0)	0 (0)	12.50 (21.65)	0 (0)	0 (0)
Puppet box	34.60 (25.09)	14.45 (13.73)	22.00 (17.14)	0 (0)	0 (0)	0 (0)	0 (0)	0 (0)
Scattered blocks	20.05 (18.34)	14.42 (9.67)	38.03 (12.86)	0.37 (0.52)	0 (0)	0 (0)	0 (0)	0.93 (0)
Wind tube experiment	4.50 (7.35)	46.29 (12.74)	31.38 (5.66)	0.40 (0.70)	0 (0)	0 (0)	0 (0)	0 (0)
Wood tree house	27.01 (35.64)	27.99 (19.68)	27.54 (19.31)	0 (0)	0 (0)	0 (0)	0 (0)	0 (0)
Xylophone	0 (0)	20 (27.39)	26.67 (25.28)	0 (0)	0 (0)	0 (0)	0 (0)	0 (0)
Scattered blocks II	6.17 (0)	8.64 (0)	12.35 (0)	0 (0)	0 (0)	46.91 (0)	0 (0)	0 (0)
Skipping pods	33.02 (40.33)	15.80 (0.59)	26.73 (10.61)	0.90 (0)	0 (0)	0 (0)	0 (0)	0 (0)

## Discussion

Our study showed how including a small scale GIS component in research focused on child play in indoor spaces can provide richer data and enhance interpretation of findings than analyzing play without positional contexts attached. Such innovative methodology helped not only to identify spaces where children played the most, but also demonstrated that changing the space layout may encourage engagement in different levels and types of physical activity and social behaviors. This key finding is meaningful and useful for programming and preschool practice.

Often, many researchers have deployed validated data collection tools on physical activity and social behaviors that do not necessarily gather information on geographical location. For instance, while the OSRAC-P coding system provides a large range of variables and categories for coding physical activity and behaviors of preschool children, this tool is not designed for geospatial analysis. Indeed, its “location” variable only indicates if play occurred in areas inside or outside the building or during the transition between inside and outside areas, which is not useful as geospatial locational information even in a small scale GIS. Also, the addition of locational information to previously collected data is rare, making the methodological contribution of this study important. To our best knowledge, our study was the first to document how to add locational information to an existing dataset with the objective of creating GIS behavior mapping. In our study, gridding and grid aggregation were done using the same videos coded with the modified OSRAC-P to provide locational information and then a GIS of play in the LTP room and, therefore, no additional data acquisition cost was required.

Our approach has many advantages over others as found in the literature. Pawlowski et al. [49], for instance, carried out a study on children’s physical activity and behavior during recess (outdoors and indoors) using a mixed-method consisting of accelerometers, GPS observations, interviews, and direct observations of play coded using the System for Observing Play and Leisure Activities (SOPLAY) and social interactions using System for Observing Children’s Activity and Relationships during Play (SOCARP). Although they produced a map showing GPS positions and the corresponding levels of physical activities, the locational data they collected with the GPS was not merged with the SOPLAY coded observations given methodological difficulties in matching GPS measurements with the SOPLAY activities of the children. Jankowska et al. [50] presented a framework for integrating GPS data with other technologies such as accelerometers for the creation of dynamic representations of physical activities and sedentary behaviors. They noted that the GPS can be unreliable sometimes, creating problems with large margins of missing GPS data.

On the other hand, video-based gridding as a method for providing locational data affords the researcher the opportunity to identify environmental information that may be missed by remote positioning during coding as it involves manual location detection at the same intervals as other variables such as physical activity. This is the case if other play variables were coded from videos and not real time. Unlike other positioning methods, video-based gridding allows the addition of locational information even after data collection and bypasses the need to ensure satellite signals are strong and not inhibited by walls and roofs during observations.

Analyzing recorded videos and physical validation of the layout map of the LTP room as seen on these videos ensured that the grids chosen were adequate for the LTP room. It also allowed all observed monthly changes in the positions of play equipment to be taken into account when positioning children in the room. That is particularly relevant for research analyzing spaces that are constantly modified, such as preschool rooms, to encourage engagement in different behaviors. The video-based gridding, therefore, is more advantageous over the GPS in addressing the changes in the utilization of the play space. More cameras will ensure that all children are seen on at least two overlapping camera views during each observation interval. Floor and wall markers (ground control points) at specific distances can be provided in indoor spaces prior to recording videos of free play for behavior mapping. These will serve as validation points to improve the accuracy of location coding with gridding.

The same set of video cameras were re-used for data collection at the 3 preschool centres covered in the previous phase of this project. Having different cameras positioned at the 3 preschool centres and at fixed locations throughout the duration of the data collection phase may increase the number of intervals collected for each preschool facility.

The changing locations and layout of equipment in the room made it impossible to use the same uniform grid aggregation scheme for equipment or play areas monthly. When the room layout included skipping pods or scattered blocks, moderate/vigorous physical activity levels were higher than observed around most fixed play equipment except the wind tube experiment. Analyzing the use of equipment and play areas in the room showed how children utilized the same types of portable play equipment differently at different areas. For example, children who played with large blocks near the horses used them differently from those who played with blocks in the open space in the middle of the room by rocking them like those who played with the horses. The changes in equipment layout monthly provides an opportunity to explore how different layouts may influence play activities and behaviors differently in the LTP room, which would greatly enhance investigations into how environments promote or inhibit particular behaviors in children [5]. Such investigations using longitudinal data over a longer period of time will also show how fixed or portable play equipment influence physical activities in preschool children may provide further insights into how to encourage children to get more physically active in their play spaces.

Many behavior maps use a variety of individual point symbols to represent observed activities or behaviors and their locations [5, 7, 49, 51]. This creates clusters of different overlapping points on the behavior maps. Using grid-based pie charts tidies up the information presented on the GIS behavior maps so that they are easier to interpret. The bubble pie charts showed the differences in categories even when points overlapped and reduced the number of symbols required to represent activities on a map. However, the large number of bubble pie charts made it harder to determine how children interacted with major play equipment or areas in the room. This was resolved by grid aggregation using major play equipment or areas observed from the videos monthly which provided insights into the location of activities and how popular different play areas and equipment were with the children in the LTP room.

For any research involving children, parental consent and children’s assent need to be collected to ensure that children’s rights are not violated during data collection [52]. Ethical issues may arise from parents not granting consent for their children to participate in studies, or when children do not themselves want to participate. For example, when a parent declines a request for a child to participate, the child may have to play in a separate play space during the video recording sessions [36]. In such cases, the parent had the right to decline, but the child who has to play alone despite wanting to play with peers may feel segregated. We prepared for these kinds of situations in our study by discussing with program staff and co-creating parallel activities for children who could not participate, involving similar activities, but out of camera view. In another example, parents may grant consent initially and then withdraw this after video collection [36]. New challenges then arise with techniques for isolating and eliminating these children to protect their privacy before any analysis can be done, which can be time consuming and difficult. Given these potential analytic and technical challenges, our study gave parents a pre-determined period of time (i.e., 60 days post data collection period) to withdraw their consent if desired. This retained their ability to shape their children’s involvement and also prevented undue impact on the data analysis stage of the project. Further, the videos analyzed for this research will remain unavailable to the public to protect the privacy of all children involved. Publishing the outcomes of research like ours where the identities of children remain hidden, will help allay the fears of parents who believe their child’s privacy may be violated and that they may be victimized by what is recorded on videos in future if they grant consent to participate.

An emerging alternative to the video coding methods applied in this research involves the application of Artificial Intelligence (AI). AI applications have been successfully used in research such as gesture pattern recognition and human physical activity recognition from smart devices [53, 54]. However, they could be very cost intensive in public health applications [55] especially for small scale applications. Further, the use of AI raises ethical concerns regarding privacy and confidentiality, particularly in research with children as advances in AI require sharing massive amount of data [56]. AI is also time intensive in the algorithm development phase as it requires a great deal of training and retraining of the algorithm required using a vast array of data to detect and classify different movements and gestures. Even in the best of studies involving AI, predicting emotions based on facial movements (one sub group of human movements) is still complex [57]. Despite its flaws, AI is a recognized technology for extracting the facial patterns of missing or wanted persons from a database and then identifying likely matches of such faces [58]. It remains an amazing advancement in technology but still presents several limitations with identifying and distinguishing humans in many cases [59]. AI has the ability to detect a wide range of human behaviors but would require a lot of supervised training on the reflex of the individuals being studied and what they mean. Therefore, AI is a very expensive technology for use in researches like ours which are not purely technology driven. With the appropriate scale of funding in future, we could evaluate the ability of AI to code the physical movements and social behaviors of preschool children after adequate supervised training.

In future, the methods described in this paper will be used to analyze preschool children’s free play in the three Strathcona County study sites over a longer period of time. Inclusion of other spaces and longer time series will afford us the opportunity to carry out more analysis on the impacts of different room layouts on interactions and play activities of children in this indoor play space. These methods can also be applied to other areas of social and behavioral research using remote video cameras for data collection, such as the utilization of public spaces including bus stations, visits to museums or art galleries (e.g., the difference in art people prefer), and food purchase.

## Conclusions

Extending the OSRAC-P tool through the addition of indoor geolocational information to other free play variables in any space provides the opportunity to infer what type of play equipment or room layouts may encourage higher levels of play activity and different social behaviors among preschool children. For example, our results showed that associative play was the main social behavior that occurred around the grocery till (fixed play equipment). However, more play intervals occurred around the skipping pods than around the grocery till. This kind of nuanced information can be valuable for program managers and decision-makers interested in offering programs and play spaces associated with targeting and strengthening particular developmental domains among children.

This research has shown an affordable method of providing locational information for behavior mapping in indoor environments alongside the modified OSRAC-P coding system. This video-based gridding has the advantage of being ideal for adding locational information to behavioral data even after video data collection has occurred. This method could be used with other systems of observing play activities and social behaviors in both children and adults. In addition to being cost-effective in indoor spaces, this gridding method allows behavior mapping without exposing children to any indoor radio frequencies which may be harmful to them [18, 19]. This gridding approach also allows for the creation of locational data for small indoor spaces where it may be unrealistic or impossible to use positioning systems such as the GPS. Finally, the methods presented could provide decision-makers with detailed information about how to design programs and play spaces to enhance children’s play experiences and increase physical activity levels, positively impacting their health.

## Data Availability

Video data used in this research are not publicly available to ensure that the identity of underage participants remains confidential and secure.

## Appendix

See Table 6.

**Table 6:** Modified OSRAC-P categories of social participation and their descriptions

Social participation type	Description
Active conversation with peer(s)	Verbal communication with another peer
Active conversation with teacher(s)	Verbal communication with teacher(s)
Aggression	Non-playful aggressive or violent behavior towards another person or an object (kicking an object out of frustration)
Associative play	A group of children engage in similar or identical activities without formal organization, the child is interested in the group playing but not in coordinating their activities with those people, or when there is no organized activity at all (substantial amount of interaction involved, but the activities are not in sync)
Can’t tell	Unable to observe on video or cannot tell
Cooperative play	The child is interested both in the people playing and in the activity they are doing, activity is organized, and participants have assigned roles (increased self-identification with a group and a group identity may emerge), dramatic play activities with roles like playing school, or a game with rules, such as freeze tag
Imitation	The child is observing and replicating another’s behavior
Onlooker behaviour	The child watches but does not get involved with an activity, may offer comments or laugh with other children but does not engage in actual activity
Parallel	Child plays beside, or in the company of, other children but does not play with peers, child plays independently with activities bringing him/her within one meter of other children sometimes (parallel play is coded regardless of the distance between the focal child and the other children if the child is attentive to peers while playing independently), child observed often playing with toys similar to those that the children around him/her are using, child is somewhat aware of, and attentive to, his/her playmates
Solitary	The child is seen playing alone at a distance from other children, child plays apart from other children at a distance greater than one meter usually with toys different from those other children are using, child is centered on his/her own activity and pays little or no attention to any other child in the area
Unoccupied behaviour	The child does not show focus or intent (e.g. child staring blankly into space, wandering with no specific purpose, or only slightly/not interested in ongoing activities), child is engaged in a functional activity such as twisting hair or fiddling with an object but is not concentrating on the activity
